# Flotation Assembly of Large-Area Ultrathin MWCNT Nanofilms for Construction of Bioelectrodes

**DOI:** 10.3390/nano7100342

**Published:** 2017-10-21

**Authors:** Andrew J. Gross, Jules L. Hammond, Michael Holzinger, Serge Cosnier

**Affiliations:** 1 Department of Molecular Chemistry, UMR CNRS-UGA 5250, Université Grenoble Alpes, 38000 Grenoble, France; andrew.gross@univ-grenoble-alpes.fr (A.J.G.); jules.hammond@univ-grenoble-alpes.fr (J.L.H.); michael.holzinger@univ-grenoble-alpes.fr (M.H.); 2 Université Grenoble Alpes, CERMAV, 38000 Grenoble, France

**Keywords:** multi-walled carbon nanotubes, optically-transparent electrode, photobioelectrode, electropolymerization, flotation assembly, nanostructured electrode, biosensor, biofuel cell

## Abstract

We report a simple, versatile, and rapid method for the fabrication of optically-transparent large-area carbon nanotube (CNT) films via flotation assembly. After solvent-induced assembly, floating films were transferred to a flat supporting substrate to form conductive and transparent CNT film electrodes. The resulting electrodes, with uniform 40 ± 20 nm multi-walled CNT (MWCNT) layers, were characterized by electrochemical and microscopy methods. The flotation method does not require specialized thin-film instrumentation and avoids the need for surfactants and pre-oxidized CNTs which can hamper electrochemical performance. A proof-of-concept nanostructured bioelectrode demonstrating high sensitivity for glucose was developed with an electropolymerized poly(pyrene-adamantane) layer for host–guest immobilization of active β-cyclodextrin tagged GOx enzymes. The polymer provides pyrene groups for cross-linking to CNTs and pendant adamantane groups for binding the β-cyclodextrin groups of the tagged enzyme. This demonstration offers a new approach for the preparation of stable and transparent CNT film electrodes with attractive electrochemical properties towards future photobio- and bio-electrochemical fuel cells, electrochemical sensors, and electroanalysis.

## 1. Introduction

The use of carbon nanotubes (CNTs) has garnered plenty of interest within the nanoscience community for interfacing soft biological systems due to their chemical stability, high electrical/thermal conductivity [1], appealing mechanical properties [2] and biocompatibility [3]. The high specific surface area (up to 850 m^2^·g^−1^) [4] and unique geometry make multi-walled CNTs (MWCNTs) an ideal candidate for the construction of highly-porous three-dimensional electrodes, especially given the ability to attach a plethora of functionalities [5,6,7]. CNT electrodes can provide a powerful platform for the immobilization of enzymes with high biomolecular activities [8,9] and have consistently demonstrated excellent performance for the electrical wiring of enzymes via direct and mediated electron transfer processes [10,11]. Three-dimensional electrodes with film thicknesses on the millimeter scale are commonly employed for enzymatic bioelectrocatalysis, but are known to have slow mass transport and ohmic resistances [12]. Use of nanoscale thin films can enhance mass transport of substrates/products and facilitate fast electron transfer with immobilized enzymes, opening up the possibility to improve bioelectrode performance for biofuel cell and biosensor devices [10,13,14]. Furthermore, CNT films with sub-100 nm thicknesses can exhibit high optical transparency which could be exploited for light-driven photoelectrochemical devices [15,16].

At present there exist several methods for producing CNT structures, such as arc discharge [17], chemical vapor deposition (CVD) growth [18], and laser ablation [19]. Whilst CVD growth offers high-quality and large-area processing, the method can yield contaminants that can adversely affect their properties, and require further processing such as thermal annealing and/or chemical treatment for their removal [20]. Furthermore, film transfer onto target substrates is impaired by hard metal catalyst etching and polymer adhesive residues [21]. With film transfer acting as the bridge between production and application, it currently presents a major challenge for successful commercialization of CNTs [22].

Solution processing of CNT-based films, therefore, offers great potential for the cost-effective preparation of devices such as flexible electrodes, supercapacitors, biosensors, and biofuel cells [23,24,25,26]. For the construction of bioelectrodes, the simple drop-casting of CNTs from aqueous and non-aqueous suspensions represents the most commonly employed method [7,27]. However, such methods are not suitable for controlling film thickness at the nanoscale. Harsh acid treatments and surfactants are also typically employed, which can hamper electrical conductivity and introduce undesired contaminants [28,29]. Here we demonstrate a new method for the preparation of stable nanoscale-thin multi-walled carbon nanotube film electrodes via rapid surfactant-free flotation assembly. As a proof-of-concept we use a Pt-MWCNT electrode to construct a biointerface for glucose detection via electro-oxidative attachment of pyrene-adamantane (pyAd) and host–guest immobilization of β-cyclodextrin (β-CD) modified glucose oxidase (GOx).

## 2. Results

### 2.1. Flotation Assembly of Thin and Thick MWCNT Films

**Figure 1:**
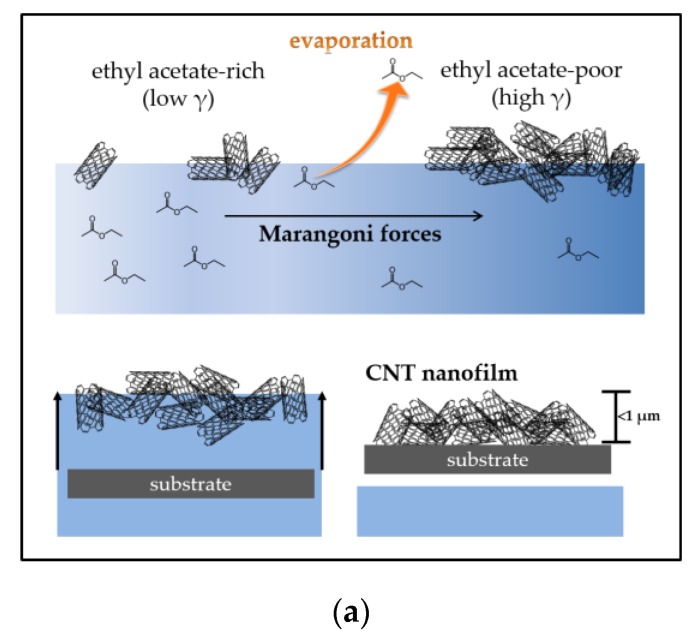

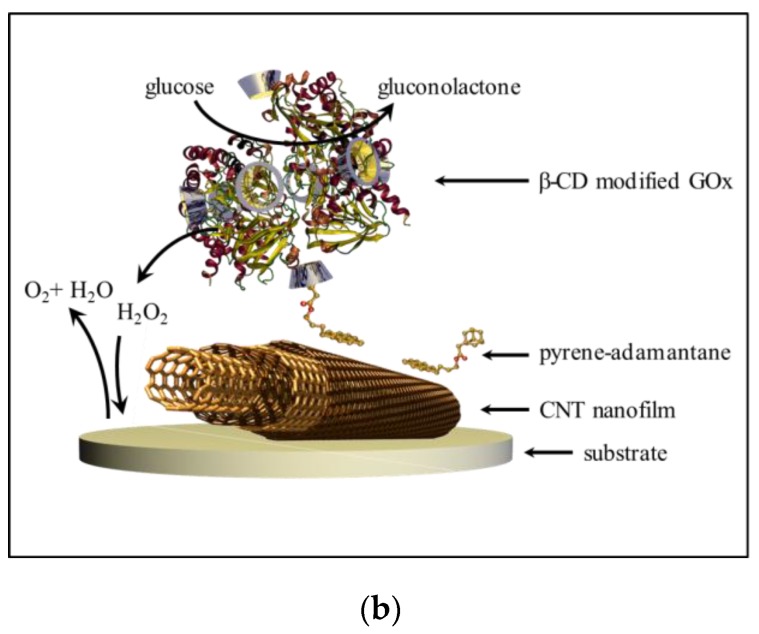
Diagrams showing: (**a**) method of flotation assembly followed by transfer onto an electrode substrate; and (**b**) constructed enzymatic interface consisting of Pt-MWCNTs/pyAd/β-CD-GOx.

Thin and thick large-area MWCNT nanofilms were formed by flotation assembly before transferring to the target substrate, as illustrated in Figure 1a. Two methods of film assembly are reported, Method A, where ethyl acetate (EA) was added to MWCNTs in aqueous solution, and Method B, where CNTs were added to EA in aqueous solution (see Section 4.2.1). For flotation assembly, the spontaneous migration of MWCNTs toward the liquid surface is attributed to Rayleigh–Bénard convection caused by evaporative cooling of the EA layer [30]. The inhomogeneous EA distribution at the liquid surface causes surface tension gradient-induced instability and results in lateral assembly of the MWCNTs through Marangoni forces parallel to the interface. The primary factors for controlling film thickness are the rate of assembly, governed by the evaporation of the solvent (ethyl acetate), and the uniformity and concentration of the CNT dispersion at the surface. To the best of our knowledge, this is the first time that a flotation assembly method has been used for the construction of carbon nanotube electrodes and bioelectrodes.

### 2.2. Surface Microscopy of Thin and Thick MWCNT Films

**Figure 2:**
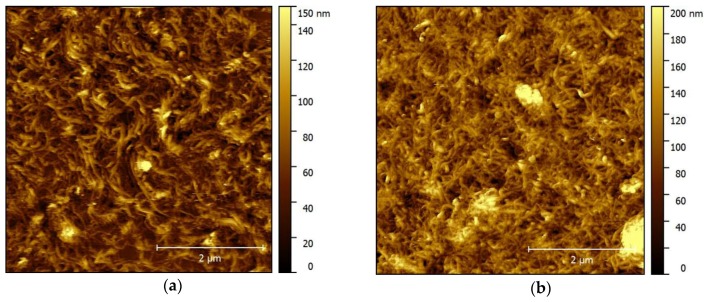

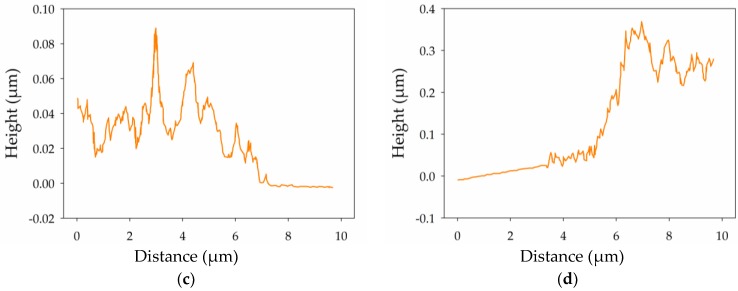
Atomic force microscopy topographic and depth profiling data for (**a**,**c**) thin and (**b**,**d**) thick MWCNT films transferred onto silicon; (**a**,**b**) 5.0 µm × 5.0 µm height topography images and (**c**,**d**) average line plots for 1.0 µm × 9.5 µm cross-sections showing film height at a boundary region.

Atomic force microscopy (AFM) was used to characterize the thin and thick film assemblies prepared by Method A (solvent to CNTs–water) or Method B (CNTs to solvent–water), respectively. Characterization was performed on film assemblies transferred onto silicon substrates due to its atomic flatness. Figure 2 shows the topographic and depth profiling plots for the (a,c) thin and (b,d) thick film assemblies. The topographic images show the formation of homogeneous networks of randomly inter-connected carbon nanotubes. The thin film shows evidence for a looser and more porous network but, overall, the two types of film are very similar. The average line plots also reveal evidence for a gradient in film thickness over a distance of a few microns at the fringes of the electrode surface. However, this represents only a minor proportion of the final electrode surface. Average film thickness values obtained from 1 µm × 9.5 µm cross-sections were calculated as 40 ± 20 nm and 285 ± 50 nm for the thin and thick films, respectively (see Figure S1 for topographic depth-profiling images). Average surface roughness values calculated from 1 μm × 1 μm regions were *R*_a_ = 18 ± 3 nm and *R*_a_ = 22 ± 5 nm for the thin and thick film assemblies, respectively. The roughness measurements clearly reveal similarly smooth nanostructured surfaces for the films with different thicknesses. Considering that the average diameter of the utilized nanotubes is 9.5 nm, the thin films, therefore, remarkably represent only a few layers of entangled nanotubes. Scanning electron microscopy and confocal laser microscopy images were also recorded and reveal similar topographic features (see Figures S2 and S3). These microscopy experiments confirmed the ability to assemble the CNT films and transfer them to various flat substrates (silicon, platinum, and gold) according to the methodology presented in Section 4.2.1.

### 2.3. Electrochemical Properties of Thin MWCNT Film Electrode

**Figure 3:**
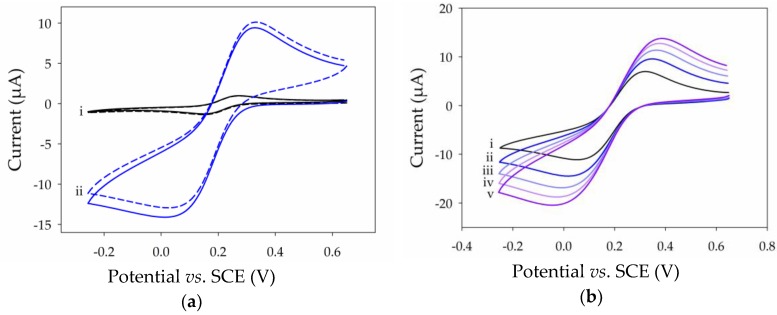
Cyclic voltammograms recorded in 1 mM K_3_Fe(CN)_6_^3−^ in 0.1 M PB pH 7 with 0.1 M KCl showing (**a**) the first two cycles at 100 m·Vs^−1^ of the (i) Pt electrode (black) and (ii) Pt-MWCNT electrode (blue); and (**b**) the first cycle at (i) 20 m·Vs^−1^, (ii) 40 m·Vs^−1^, (iii) 60 m·Vs^−1^, (iv) 80 m·Vs^−1^, and (v) 100 m·Vs^−1^.

To probe the electrochemical performance of MWCNT film modified electrodes, initial experiments were carried out with the benchmark ferri-/ferrocyanide (Fe(CN)_6_^3−/4−^) redox probe. This redox probe was chosen as it is well known to have quasi-reversible, surface-sensitive electrochemistry. A cyclic voltammogram (CV) (Figure 3a, black) recorded in 1 mM Fe(CN)_6_^3−^ in pH 7 phosphate buffer (PB) solution at a Pt electrode before MWCNT film deposition shows well-defined and chemically-reversible behavior with a peak-to-peak separation (Δ*E*_p_) of 128 mV and an anodic peak current of 1.1 µA. After transfer and drying of the floating film to Pt, the CV obtained in Fe(CN)_6_^3−^ solution (Figure 3a, blue) showed a larger Δ*E*_p_ of 313 mV and an increase in anodic peak current to 10.2 µA, consistent with successful film deposition. The resultant change in peak separation indicates that the apparent electron transfer kinetics for the redox couple are slower at the MWCNT film. However, the increase in peak currents suggests an approximate increase in the electroactive surface area by a factor of 10 simply by deposition of only a few multilayers of nanotubes. The increase in peak currents and a slightly sigmoidal peak shape is also indicative of an increased rate of diffusive mass transport. Due to the superior properties and expected thin-layer mass transport effects, it was unexpected that the MWCNT film would exhibit a larger peak separation characteristic of sluggish kinetics. Nevertheless, large Δ*E*_p_ values for the Fe(CN)_6_^3−/4−^ couple at the CNT-modified electrodes compared with traditional electrodes has previously been observed when *N*-Methyl-2-pyrrolidone (NMP) has been used as the dispersion solvent [31]. This effect is attributed to the presence of physisorbed organic NMP residues with a low dielectric constant which can inhibit electron transfer.

The stability to repeat potential cycling was also tested for the thin MWCNT film on Pt, as shown in Figure S4. Essentially, no change in the voltammetry was observed after 20 cycles in Fe(CN)_6_^3−^ solution, consistent with the stable attachment of a CNT film to the supporting Pt substrate. Anodic and cathodic peak currents were subsequently measured from CVs recorded in Fe(CN)_6_^3−^ solution (Figure 3b) and varied linearly with the square root of the scan rate (see Figure S5) according to the Randles-Sevcik equation, consistent with the quasi-reversible diffusional behavior expected at a standard electrode.

**Figure 4:**
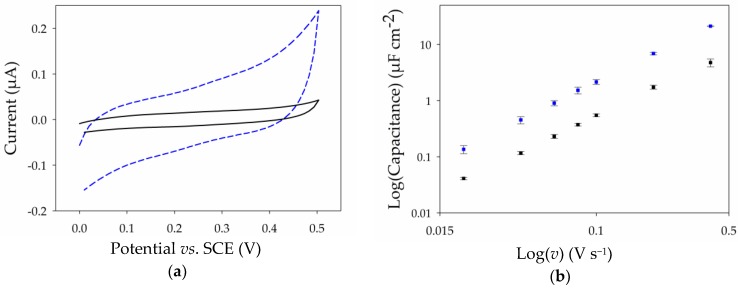
Electrochemical characterization of a planar Pt electrode (solid, black) and a Pt-MWCNT electrode (dash, blue) fabricated using Method A: (**a**) cyclic voltammograms recorded at 100 mV·s^−1^ in 0.1 M PB at pH 7 with 0.5 M KCl; and (**b**) log-log plot of the capacitance versus scan rate for the Pt electrode (black) and Pt-MWCNT electrode (blue).

The voltammetric response of both the Pt and Pt-MWCNT electrodes was also recorded in 0.1 M PB at pH 7 with 0.1 M KCl as supporting electrolyte to provide an insight on the capacitance of the thin film MWCNT electrodes versus that of the Pt electrode (Figure 4a). Capacitance values were estimated based on geometric area for a range of scan rates between 10 and 400 mV·s^−1^ and are presented in Figure 4b. The larger capacitances at the CNT film electrode further demonstrate an increase in surface area following modification, while the relatively low values of capacitance per area further indicate the formation of a MWCNT nanofilm consisting of just a few entangled nanotubes [32]. These values are, nevertheless, comparable with previously-reported values for CNTs [33]. For electroanalysis and many sensors, this low capacitance mitigates masking of the analyte signal from non-Faradaic charging of the electric double layer.

### 2.4. Surface Modification of Thin MWCNT Film Electrode with Pyrene-Adamantane

**Figure 5:**
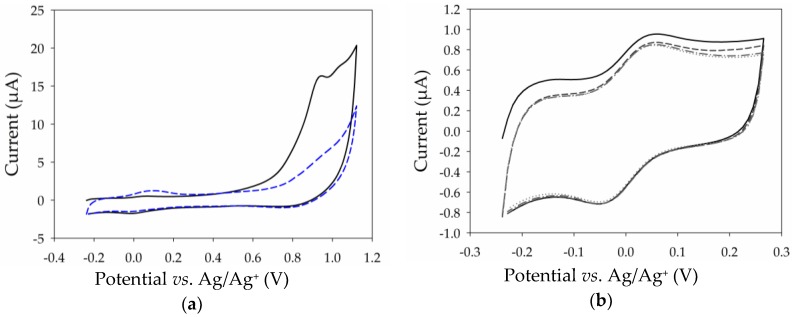
Cyclic voltammograms recorded at 100 mV·s^−1^ in CH_3_CN with 0.1 M LiCLO_4_ showing (**a**) first (solid, black) and second (dash, blue) cycles of Pt-MWCNT after immersion in pyrene-adamantane solution; (**b**) 1st, 2nd, 10th, and 20th (solid, dash, dash-dot, and dot, respectively) cycles of immobilized poly(pyrene) redox groups after electropolymerization of pyrene-adamantane.

Towards the development of a nanostructured biointerface, we investigated the possibility to modify the CNT film electrode by electropolymerization to introduce chemical functionalities for enzyme attachment. To this purpose, we electropolymerized a pyrene-adamantane derivative that we developed previously for functionalization of drop-casted single-walled carbon nanotube film electrodes [34]. Here, the fabricated MWCNT electrode is first immersed in acetonitrile (CH_3_CN) containing 2 mM pyrene-adamantane for 30 min to adsorb the modifier to the nanotubes by π–π stacking of pyrene groups with nanotube sidewalls. The electrode is then rinsed in CH_3_CN and modified via oxidative electropolymerization by recording two consecutive cyclic voltammograms in CH_3_CN containing 0.1 M LiCLO_4_. It is noted that no evidence for film delamination was observed after immersion and rinsing of the electrode in organic solvent, providing further support for the stable attachment of the CNT films to the surface. Films previously reported by drop-casting of CNTs on electrode surfaces show similar resistance [34] with the stability, depending on the CNT dispersion and sufficient drying of the CNT layer. Figure 5a shows a representative example of the first and second cycles recorded between 0.0 and 1.1 V *vs*. Ag/Ag^+^ in the monomer-free solution.

On the first CV cycle, an irreversible peak at 0.95 V is observed, attributed to the electro-oxidation of the pyrene monomer to its cationic radical [35]. On the second scan, the disappearance of the large oxidative peak at 0.95 V and the appearance of a very well-defined symmetric redox couple centered at *E*_1/2_ = 0.0 V *vs*. Ag/Ag^+^ is observed, characteristic of the formation of an electrogenerated poly(pyrene) film on the surface. Potential cycling was subsequently performed for 20 cycles at 20 mV·s^−1^ between −0.25 and 0.2 V *vs*. Ag/Ag^+^ and revealed excellent stability of the electroactive polymer backbone (Figure 5b). This redox couple with a Δ*E*_p_ of 96 mV is much better resolved and more stable than that observed in our previous work on drop-coated SWCNT film electrodes [34], highlighting the excellent properties of the ultrathin film MWCNT electrodes. Next, the surface concentration of immobilized electroactive pyrene groups was estimated from the anodic wave of the second cycle and estimated to be (8.4 ± 1.2) × 10^−10^ mol·cm^−2^ (*n* = 5), according to Equation (1), where *Q* is the integrated charge, *n* is number of moles, *F* is the Faraday constant, and *A* is the geometric surface area. The estimated surface concentration is comparable to that observed previously at the Pt-SWCNT electrodes despite the use in this work of fewer electropolymerization cycles and the ultrathin CNT network [34]. The ability to achieve high electroactive surface coverage with only two polymerization cycles is attractive for mitigating the generation of a thick, hydrophobic, and poorly conducting polymer which can hinder electrolyte and product/reactant diffusion, as well as electrical conductivity.

(1) $$\Gamma \;=\;Q\cdot n^{-1}\cdot F^{-1}\cdot A^{-1}$$

### 2.5. GOx-Modified Thin MWCNT Film Electrode

To demonstrate the utility of the MWCNT nanofilms for construction of a biointerface, we prepared bioelectrodes functionalized with β-cyclodextrin-tagged glucose oxidase (β-CD-GOx) and explored the amperometric detection of glucose. The modified glucose oxidase was immobilized on poly(pyrene-adamantane) modified electrodes via drop-casting for 30 min. The specific enzyme immobilization is based on the affinity system between adamantane and β-cyclodextrin, which forms a 1:1 inclusion complex with a high binding constant typically between 1 × 10^4^ M^−1^ and 1 × 10^5^ M^−1^ [36]. After modification with enzyme and thoroughly rinsing with phosphate buffer, the bioelectrode was examined for glucose detection via chronoamperometry.

**Figure 6:**
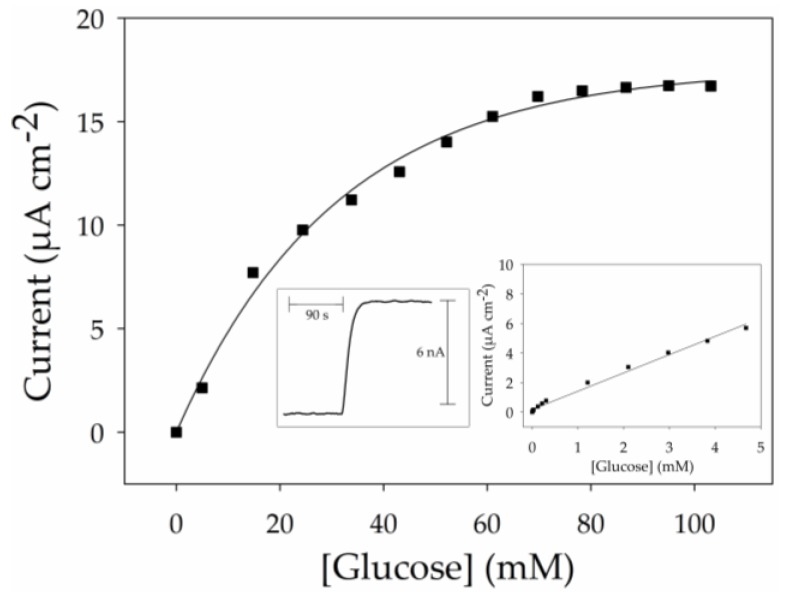
Response for amperometric glucose detection in 0.1 M PB at pH 7 for the range 5 to 110 mM. Inset, left: typical amperometric response obtained at 5 mM. Inset, right: linear calibration plot for the range 1 µM to 5 mM in 0.1 M PB at pH 7. Data recorded at 0.6 V *vs*. SCE at room temperature without stirring.

The biosensor system is based on the enzymatic oxidation of glucose with the concomitant production of hydrogen peroxide in the presence of dissolved oxygen [13]. The enzymatically-generated hydrogen peroxide is then detected by electrochemical oxidation at a fixed potential of 0.6 V *vs*. saturated calomel electrode (SCE). Performance of the biosensor was examined for successive injections of glucose for the concentration range of 1 µM to 110 mM in stirred 0.1 M phosphate buffer at pH 7 at room temperature. Figure 6 shows the anodic current response of the bioelectrode as a function of glucose concentration corroborating the expected anchoring of β-CD-GOx onto the functionalized MWCNT nanofilm. The average response time of the biosensor (determined as the time required to reach a new current value indistinguishable from the final steady-state current, after a glucose injection) was 24 s for the concentration range of 1 µM to 5 mM. An example of the steady-state current response obtained at 5 mM glucose concentration is presented in the left inset of Figure 6. The calibration curve shows the development of a hyperbolic plot which reaches a current plateau at a saturating glucose concentration of 90 mM. The glucose sensitivity (1.41 mA·M^−1^·cm^−2^) was determined from the slope of the initial linear part of the calibration curve. It should be noted that this sensitivity value is 44% higher than those previously reported for a bioelectrode based on a poly(pyrrole-biotin) film modified by the same enzyme type, β-CD-GOx [37]. This illustrates the higher permeability of the modified MWCNT nanofilm compared to an electrogenerated organic polymer. The apparent Michaelis–Menten constant (K_M_) is calculated using *I*_max_ = 16.7 µA·cm^−2^ as 6 mM for the linear region in Figure 6. This value is attractively smaller than those previously reported for GOx-modified CNT electrodes [34,38], reflecting the absence of steric constraints towards the permeation of glucose, with the enzymatic reaction being limited by the oxygen concentration. This can be ascribed to the highly permeable structure of the ultrathin 3D-structured MWCNT/pyAd matrix which facilitates fast transport of oxygen and hydrogen peroxide at the electrode. The low K_M_ may also be a result of the high degree of freedom of enzymes immobilized by a single point of attachment and their proximity to the electrode sensing surface.

## 3. Discussion

In summary, we report a simple and rapid flotation method for the assembly of as-prepared non-functionalized carbon nanotubes into nanoscale films with different nanoscale thicknesses. The assembly is performed only in solvents and does not require surfactants, physical deposition, or templating. Significantly, very thin sub-50 nm films equivalent to only a few layers of carbon nanotubes can be prepared and easily transferred to flat surfaces, potentially including flexible and convex structures. Optical and electrochemical performance reveals the formation of homogeneous 3D-structured nanofilms with enhanced surface area, stability, low capacitance, and excellent electrochemical behavior. Finally, we demonstrate how these very thin films can be used for electropolymerization and host-guest immobilization of an enzyme for a proof-of-concept glucose biosensor with satisfactory performance. The fabrication method developed here introduces flotation assembly as a new method for construction of nanoscale-thin transparent CNT film electrodes and bioelectrodes with great potential in bioelectrocatalysis and potentially exciting possibilities for photobioelectrocatalysis. The formation of transparent conducting CNT electrodes also holds promise in the development of optoelectronic and photovoltaic devices. Future investigations are now required to optimize film assembly, such as: (i) controlling the ambient temperature to change the solvent-aqueous interface temperature differential; (ii) adjusting the solvent volume; (iii) changing container dimensions; (iv) using different types and concentrations of CNT dispersions; and (v) changing the solvent (such as using diethyl ether). In this way, highly reproducible films with tunable properties, including fast electron transfer kinetics and optical transmittance, can be prepared for the target application.

## 4. Materials and Methods

### 4.1. Materials

Monosodium phosphate monohydrate (NaH_2_PO_4_, ≥98%), disodium hydrogen phosphate heptahydrate (Na_2_HPO_4_, 98–102%), acetonitrile (CH_3_CN, ≥99.5%), ethyl acetate (EA, ≥99.5%), *N*-Methyl-2-pyrrolidone (NMP, ≥99%), d-(+)-glucose (≥99.5%), potassium chloride (KCl, ≥99%), potassium ferricyanide (K_3_Fe(CN)_6_, ≥97%), and glucose oxidase (GOx from *Aspergillus niger*, 179 U·mg^−1^) were all purchased from Sigma-Aldrich (Sigma-Aldrich Co., St. Louis, MO, USA) and used as received. Lithium perchlorate (LiClO_4_, ≥99%) was obtained from Acros Organics (Acros Organics BVBA, Geel, Belgium) and used as received. Commercial-grade multi-walled carbon nanotubes (MWCNTs, Ø = 9.5 mm, 1.9 µm length, ≥95% purity) were obtained from Nanocyl (Nanocyl SA, Sambreville, Belgium) and used as received without further purification. Synthesis of pyrene-adamantane and β-cyclodextrin tagged glucose oxidase is described in the group’s previous work [34]. Aqueous solutions were prepared using ≥15 MΩ·cm distilled water from a Millipore (Millipore Co., Burlington, MA, USA) Ultrapure system. Enzymes were stored at −20 °C. Glucose solutions were left to mutarotate overnight to β-d-glucose prior to use.

### 4.2. Methods

#### 4.2.1. Preparation of MWCNT Films by Flotation Assembly

Two methods of electrode fabrication by flotation assembly were developed to create either thin or thick films. In Method A, a suspension consisting of 1 mL of MWCNTs (1 mg·mL^−1^) in *N*-methyl-2-pyrrolidone (NMP) is first added to the surface of a 20 mL H_2_O solution in a Petri dish, then 0.5 mL of ethyl acetate (EA) is added to the solution surface, followed by a further dropwise addition of 0.5 mL to the solution surface to complete the assembly. In comparison, for Method B, 1 mL of MWCNTs suspended in NMP (1 mg·mL^−1^) is added to a solution in a petri dish containing 20 mL of H_2_O and 0.5 mL EA, then a further 0.5 mL of EA is added dropwise to the solution surface. In both cases, after the solution is settled, the floating film is then transferred onto the target substrate simply by submerging the substrate under the film and lifting out of the solution. The electrode is then dried overnight under vacuum. The floating films are prepared in large (Ø = 8 cm) Petri dishes at room temperature in air permitting the fabrication of CNT film electrodes with lateral dimensions on the centimeter scale.

#### 4.2.2. GOx-β-CD Immobilization

The enzymatic bioelectrodes were fabricated from poly(pyrene-adamantane) modified electrodes by drop-casting 5 μL of 0.5 mg·mL^−1^ GOx-β-cyclodextrin onto the surface and allowing the enzyme to immobilize for 30 min. After thoroughly rinsing with 0.1 M phosphate buffer (PB) pH 7, then carefully drying with a stream of nitrogen, the analytical performance of the bioelectrode was tested for glucose determination.

#### 4.2.3. Electrochemistry

Cyclic voltammetry experiments were performed at room temperature with an Eco Chemie Autolab potentiostat with GPES 4.9 software (Metrohm AG, Herisau, Switzerland). A saturated calomel electrode (SCE) or Ag/AgCl (sat. KCl) was used as the reference electrodes for aqueous electrochemistry, together with a Pt wire counter electrode and the working electrode (Pt or Pt-MWCNT with Ø = 0.7 mm) in the classical three-electrode cell configuration. For non-aqueous electrochemistry an Ag/Ag^+^ (AgNO_3_, 10 mM in CH_3_CN + 0.1 M LiClO_4_) reference was used. Amperometric experiments were carried out with mild stirring (<250 rpm) at room temperature with a Tacussel PRG-DL potentiostat (OrigaLys Electrochem SAS, Rillieux-la-Pape, France) with an E-recorder interface and E-chart software (eDAQ Pty Ltd., Sydney, NSW, Australia).

#### 4.2.4. Microscopy and Spectroscopy Imaging

Scanning electron microscopy (SEM) images were recorded using a FEI/Quanta FEG 250 scanning electron microscopy (Thermo Fisher Scientific Co., Waltham, MA, USA) operating with an accelerating voltage of 2 kV without metal coating. Laser microscopy images were recorded using a Keyence VK-X200 laser scanning confocal microscope (Keyence Co., Osaka, Japan). Atomic force microscopy (AFM) measurements were performed on a Si(100) substrate using a Dimension Icon (Bruker, Billerica, MA, USA) with SCANASIST-Air probes in peak-force mode and processed using Gwyddion 2.41 software (Czech Metrology Institue, Brno, Czech Republic).

## Supplementary Materials

The following are available online at http://www.mdpi.com/2079-4991/7/10/342/s1, Figure S1. AFM images; Figure S2. SEM images; Figure S3. Laser microscopy images; Figure S4 and Figure S5. Electrochemical data.
