# Supplementary material for: Flotation Assembly of Large-Area Ultrathin MWCNT Nanofilms for Construction of Bioelectrodes
Source: Nanomaterials (Basel). 2017 Oct 21;7(10):342. doi: 10.3390/nano7100342 (PMC5666507; doi:10.3390/nano7100342)
Supplement: Supplementary file 1 [file nanomaterials-07-00342-s001.pdf]

Flotation Assembly of Large-Area Ultrathin MWCNT Nanofilms for Construction of Bioelectrodes

Andrew J. Gross [1,2], Jules L. Hammond [1], Michael Holzinger [1] and Serge Cosnier [1,*]

[1] Department of Molecular Chemistry, UMR CNRS-UGA 5250, Université Grenoble Alpes, 38000 Grenoble, France; andrew.gross@univ-grenoble-alpes.fr (A.J.G.); jules.hammond@univ-grenoble-alpes.fr (J.L.H.); michael.holzinger@univ-grenoble-alpes.fr (M.H.)
[2] Université Grenoble Alpes, CERMAV, 38000 Grenoble, France
* Correspondence: serge.cosnier@univ-grenoble-alpes.fr; Tel.: +33-4-56-52-08-10

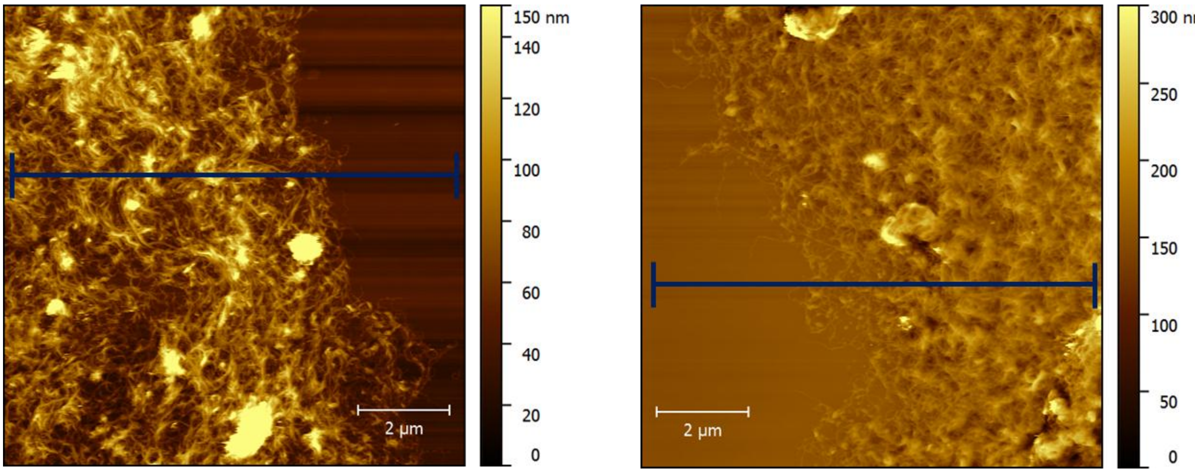

(**a**) (**b**)

Figure S1. Atomic force microscopy topographic images (10.0 µm × 10.0 µm) for depth profiling recorded at (**a**) thin and (**b**); blue markers indicate the 1.0 µm × 9.5 µm cross-sections corresponding to the average line plots in **Figure 2c** and **Figure 2d**.

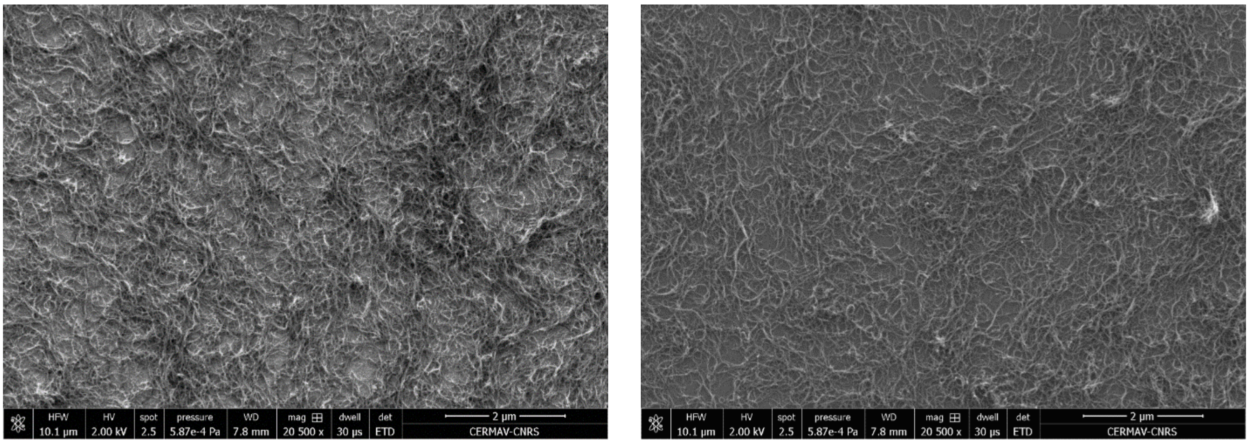

(**a**) (**b**)

Figure S2. Scanning electron microscopy images showing (**a**) thin MWCNTs transferred to a Au substrate; (**b**) thin MWCNTs transferred to a Si substrate.

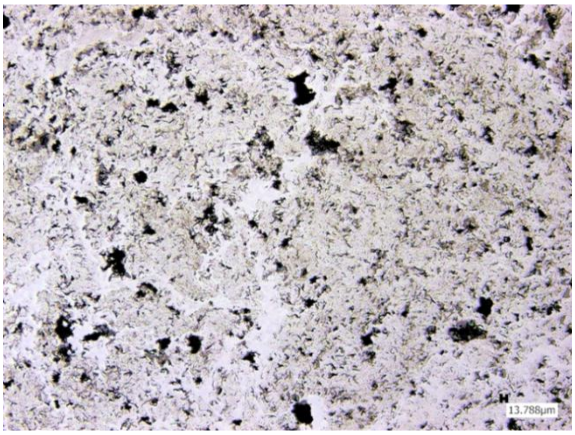

(**a**)

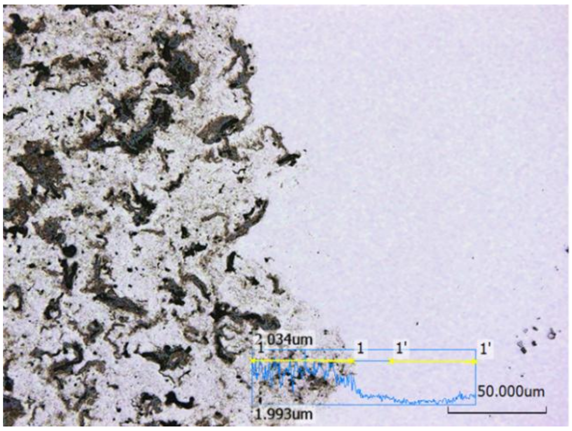

(**b**)

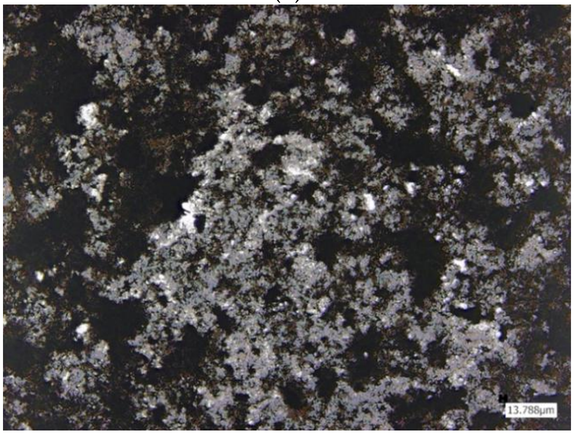

(**c**)

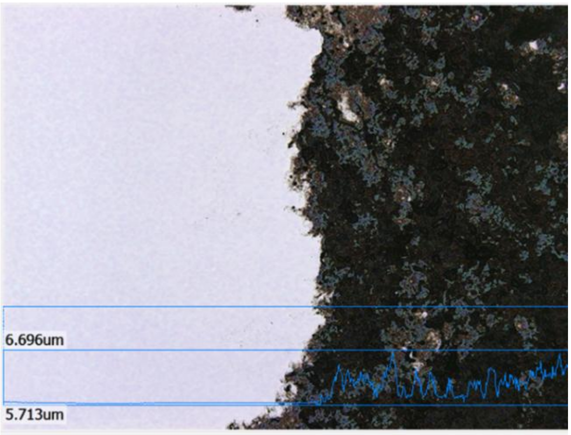

(**d**)

Figure S3. Confocal laser images of (**a**,**b**) thin MWCNTs transferred to a Si substrate: (**a**) typical central area and (**b**) edge boundary with height measurement; (**c**,**d**) thick MWCNTs transferred to a Si substrate: (**c**) typical central area and (**d**) edge boundary with height measurement.

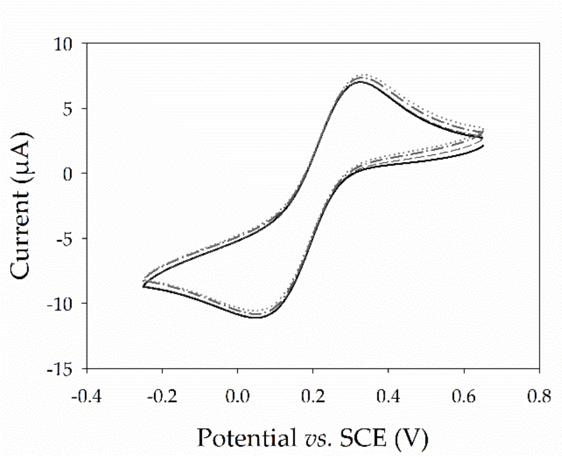

Figure S4. Cyclic voltammograms recorded at thin MWCNT on Pt in 1 mM $K_3Fe(CN)_6^{3-}$ in 0.1 M PB pH 7 with 0.1 M KCl as supporting electrolyte showing 1st, 2nd, 10th and 20th (solid, dash, dash-dot and dot, respectively) cycles.

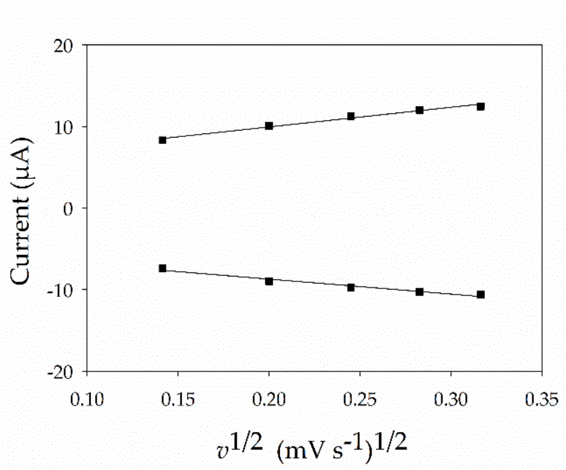

Figure S5. Linear dependence (R^2 = 0.980) of peak current versus scan rate for the anodic and cathodic peaks at thin MWCNT on Pt in 1 mM $K_3Fe(CN)_6^{3-}$ in 0.1 M PB pH 7 with 0.1 M KCl.
